# Neuroticism, Empathy, and Internet Addiction in Different Roles in Cyberbullying

**DOI:** 10.1192/j.eurpsy.2022.698

**Published:** 2022-09-01

**Authors:** G. Soldatova, S. Chigarkova

**Affiliations:** Lomonosov Moscow State University, Faculty Of Psychology, Moscow, Russian Federation

**Keywords:** neuroticism, Empathy, internet addiction, cyberbullying

## Abstract

**Introduction:**

Research on cyberbullying has focused on the psychological characteristics of victims and aggressors, but the important roles of bystanders and defenders have not been sufficiently explored (Escortell et al., 2020; Polanco-Levican, Salvo-Garrido, 2021; Schultze-Krumbholz et al., 2018).

**Objectives:**

The aim is to compare neuroticism, empathy, and Internet addiction in adolescents in different roles in cyberbullying.

**Methods:**

1505 adolescents aged 12-17 years old from 8 Federal regions in Russia appraised their experience of cyberbullying (as aggressors, victims, passive bystanders and defenders) using vignettes and filled Aggression Questionnaire (Buss, Perry, 1992), Ten-Item Personality Inventory (Gosling et al., 2003; Egorova, Parshikova,2016); Interpersonal Reactivity Index (Davis, 1983; Karyagina, Kukhtova, 2016) and Chen Internet Addiction Scale (in adaptation Malygin, Feklisov, 2011).

**Results:**

More than one-third of adolescents (37%) reported experience of cyberbullying in different roles, mostly as passive bystanders (52%). Among the active roles were 30% defenders, 10% victims and 7% aggressors. Aggressors have the lowest empathy scores on the scales of Fantasy (F= 5.424, p=0.001) and Empathic Concern (F= 2.914, p=0.034) and Neuroticism (F= 3.060, p=0.028), while defenders, on the contrary, have the highest levels. The level of these psychological characteristics in victims is lower than in defenders and bystanders. These results are coherent with a number of studies (Escortell et al., 2020; Schultze-Krumbholz et al., 2018). There are no significant differences in Internet addiction between adolescents in different cyberbullying roles.

**Conclusions:**

Results can be used to effective intervention and prevention of cyberbullying based on specific personality role profiles. The research was supported by RSF (project No. 18-18-00365)

**Disclosure:**

This work was supported by the Russian Science Foundation, project # 18-18-00365.

